# Mediating engagement in a social network intervention for people living with a long‐term condition: A qualitative study of the role of facilitation

**DOI:** 10.1111/hex.13048

**Published:** 2020-03-11

**Authors:** Elizabeth James, Anne Kennedy, Ivaylo Vassilev, Jaimie Ellis, Anne Rogers

**Affiliations:** ^1^ School of Health Sciences University of Southampton Southampton UK; ^2^ NIHR CLAHRC Wessex University of Southampton Southampton UK

**Keywords:** patient facilitation, self‐management support, social context, social network intervention, social participation

## Abstract

**Background:**

Successful facilitation of patient‐centred interventions for self‐management support has traditionally focussed on individual behaviour change. A social network approach to self‐management support implicates the need for facilitation that includes an orientation to connecting to and mobilizing support and resources from other people and the local environment.

**Objective:**

To identify the facilitation processes through which engagement with a social network approach to self‐management is achieved.

**Method:**

Thematic analysis was used to analyse data from a longitudinal study design using quasi‐ethnographic methods comprising non‐participant observation, video and qualitative interviews involving 30 participants living with a long‐term condition recruited from a marginalized community.

**Results:**

Findings centred on three themes about the social network approach facilitation processes: reversing the focus on the self by bringing others into view; visualization and reflection as a mediator of positive disruption and linking to new connections; personalized matching of valued activities as a means of realizing preference elicitation.

**Discussion and conclusions:**

Engagement processes with a social network approach illuminated the relevance of cognizance of an individual's immediate social context and forefronting social participation with others as the bases of self‐management support of a long‐term condition. This differs from traditional guided facilitation of health behaviour interventions that frame health as a matter of personal choice and individual responsibility.

## 1 BACKGROUND

The onset and trajectory of a long‐term condition (LTC) often results in a retreat from activities and interactions taking place in the public sphere.^1^ People with LTCs identify loss of social contact, the ability to reciprocate and contribute to society and receipt of resources from the community and locality.^2^ The latter has contributed to the burden of illness and preventing living life as fully as possible.^3^, ^4^ A social network approach (SNA) to self‐management provides a means of mobilizing, mediating and accessing support.^5^ It enables people to access and incorporate the resources and connections that provide support for living everyday life and requires attention to be placed on the local environments as a means of engaging individuals in self‐management support (SMS) activities.^5^ Whilst guided facilitation is recognized as a necessary component of implementing patient‐centred interventions and means of engaging with patients in introducing strategies and practices of SMS,^6^, ^7^, ^8^ little attention has been given to how to effectively bring into view and facilitate elements of social context relevant to a social networked and broader orientation to SMS.

In theory, the delivery of self‐management interventions and shared decision making acknowledges the role of social context.^9^, ^10^, ^11^ In practice, the social and emotional aspects tend to be side‐lined, whilst SMS trainers' primary orientation is towards prioritizing pre‐determined health behaviour change and individuals' capacity and responsibility to initiate and sustain strategies for self‐management.^12^, ^13^, ^14^, ^15^ Research suggests a risk of creating a sense of disempowerment from a traditional, guided facilitation approach (eg behavioural activation) stemming from the need for individuals to acknowledge that they are unable to cope alone without professionals' support. This in turn risks compounding feelings of worthlessness, low mood and loss of a valued identity.^16^

By contrast, the design of facilitation in a SNA needs to reflect as a central tenet the social context and possibilities for social participation relevant to people's lives, particularly of those living in disadvantaged circumstances.^17^, ^18^ Thus, the elements and process of the successful facilitation of socially orientated and networked interventions are likely to differ in terms of processes as well as content. Building rapport and good communication skills are important pre‐requisites for facilitating any SMS intervention. However, a SNA differs in its facilitation processes in orientation towards the idea of connections and linkages based on what is familiar to people in their everyday, domestic lives. There is a need to explore and better understand the nuances of these processes and the potential for achieving engagement by focusing on what is external to the person rather than the focus being internal as part of a personalized, therapeutic process.

### 1.1 A social network intervention (SNI) and facilitation of SMS

Facilitation of a SNI centres on connecting people to and engaging them in relationships, valued activities and resources through participating in local activities.^19^ This is informed by a capabilities approach, which suggests that opportunities individuals have to undertake valued activities are shaped by interactions between individuals, their environment and in particular their social relationships and expressions of values and preferences.^20^, ^21^

### 1.2 The social network intervention

An online tool (GENIE—Generating Engagement in Network Involvement) maps social networks, helping people to select their preferences and engage with local support resources. The components of GENIE are described in Table 1.

**Table 1:** Components of GENIE

Stage	Activity	Purpose
Step 1: Mapping using concentric circles	People, places, pets and objects (social network members) are mapped onto 3 concentric circles. Different circles capture the importance of each network member in supporting long‐term condition (LTC) management, together with relationship and frequency of contact	To explore how network members, contribute to SMS in everyday life To create a visual image of existing support network To guide a conversation about extending current support and accessing new sources of support To capture change over time
Step 2: Preference questionnaire	13 online questions covering a range of local community activities and resources within the person's post‐code area	To find out what an individual enjoys doing or used to enjoy doing in the past To link relevant network members to chosen activities To select 3 most important activities in order to prevent information overload
Step 3: Linking to local activities	Intervention software selects from internal database all relevant local resources that correspond to individual's chosen activities. Information is displayed, including location of activity on Google Map	To link individuals with prioritized and valued local activities To discuss opportunities and challenges relating to attending a new activity

The role of facilitation.

1. **Personal network mapping**: A modified, hierarchical mapping technique using three concentric circles to create visual representations of personal communities.^22^, ^23^ Constructing the network involves the person and facilitator in seeking to understand the actual and potential environment that has relevance for leveraging what is beneficial to living daily life with a LTC.^24^ This requires the facilitator to place the emphasis on the participant at the centre of the circle and encourage them to think about why and how some people and resources might be more or less important to them.^25^
2. **Preference elicitation and linking to resources:** Reflecting on availability and connection to localized support and resources based on personal preferences and acceptability that provide opportunities and encouragement to engage with sustainable health choices.

Previous research suggested that GENIE worked best as a facilitated process (Kennedy, 2016), rather than being completed by an individual alone, but questions remain about the content and mechanisms of facilitation when using a SNA in a community setting. We were interested in exploring the facilitator role when taken up by lay health workers living and working in the same community setting as participants, as they were likely to be familiar with the culture and values of that locality.

## 2 RESEARCH AIMS

To explore the role of facilitation of a SNI delivered by lay health workers in a community setting.

To identify the facilitation processes through which engagement with a SNA to SMS is achieved.

## 3 METHODS

A longitudinal study design using quasi‐ethnographic methods comprising non‐participant observation, video recording and qualitative interviews. Participants (n = 30) living with a LTC were recruited from a marginalized community. Data collected (T1) comprised visually recordings of intervention delivery, observational notes and audio recordings of post‐intervention interviews. The use of videos allowed members of the research team to observe each intervention taking place, rather than a single researcher, and enabled a more accurate, nuanced and collective analysis of the facilitation process.

Follow‐up data were collected at 3 months (T2, face‐to‐face semi‐structured interviews) and 6 months (T3, telephone interviews) to capture change over time. The circle diagram captured changes over time in the position of network members on the map (Vassilev, 2018). The mapping exercise was also a heuristic device which could indicate relational shifts in people's lives over time and enabled the facilitator to elicit people's underlying rationales for these. Data collected at different time points reflected how things changed subtly, including small changes in relationships and changes in meanings of relationships.

Audio recordings were transcribed verbatim. Thematic analysis informed by framework analysis included a priori codes/categories relating to the role of facilitation and facilitation processes, alongside an inductive approach whereby coding and theme generation were directed by the content of the data. Initial coding and collating were undertaken by researcher (EJ) to identify broad patterns of meaning prior to the viability of potential themes being discussed and agreed within the team. In addition, parts of the data set were coded independently (AR/AK/IV/JE) to ensure inter‐rater reliability and coding consistency. Themes were refined and defined, including a detailed analysis of each theme to draw out key findings.

Most salient to this study was engagement in the network mapping, as an initial exercise. However, we were also interested in how people reflected over time. Engagement means participation in the mapping exercise and what it represents for people in terms of their own micro‐social world. Engagement is when people's attention is on things outside of themselves, for example thinking about the people around them and what these people do. On the participant's part, engagement also manifests in raised awareness and reflection over time of the benefits of adopting a SNA to SMS.

### 3.1 Sample and recruitment

The sample was drawn from a marginalized community, the Isle of Wight. Separated from the mainland, social, economic and political barriers contribute to the marginalization of the Island, as does an ageing, vulnerable population (27% aged 65+ years, 1 in 6 of whom live alone). The full sample took part in observations and interviews (3 participants withdrew). Table 2 shows participants' age, gender, LTC and where the intervention took place.

**Table 2:** Demographics/LTC/location

ID	Gender	Age	LTC	Location of observation and T1 interview
01	F	51	Diabetes T2	University
02	M	70	Diabetes T2, Cerebral Palsy	Home
03	F	57	Diabetes T1	Cancer support centre
04	M	54	Diabetes T2	Community library
05	M	70	Diabetes T2	Community library
06	M	68	Diabetes T2	Home
07	F	59	Diabetes T2	Community library
08	F	66	Diabetes T2	Home
09	M	43	Diabetes T2	Medical centre
10	F	66	Diabetes T2	Medical centre
11	M	75	Diabetes T2	Home
12	F	59	Diabetes T2	Community library
13	M	76	Visual impairment, diabetes T2	Home
14	M	73	Diabetes T2	Home
15	M	67	Diabetes T2	Home
16	F	50	Behcet's disease, associated arthritis	Home
17	M	70	Parkinson's, epilepsy, arthritis, depression, intestinal disorder	Home
18	F	83	Heart disease	Home
19	F	48	Multiple sclerosis	Community library
20	M	84	Parkinson's, arthritis	Home
21	F	45	Multiple sclerosis	Home
22	M	69	Heart disease, in recovery from prostate cancer	Home
23	M	67	Heart disease/heart attack, arthritis, nerve damage	Home
24	M	81	Heart disease, arthritis, diabetes T2	Home
25	M	45	Multiple sclerosis	Home
26	M	69	Atrial fibrillation, arthritis	Home
27	M	81	Heart disease, arthritis, prostate cancer	Home
28	F	56	Arthritis, depression	Home
29	F	45	Arthritis, asthma, anxiety/depression, plantar fasciitis	Home
30	F	66	Castleman's disease, diabetes, heart disease, arthritis, depression	Home

Participants were recruited via two routes. Firstly, lay health workers identified clients who matched the inclusion criteria, introduced them to the study and invited them to participate. Secondly, the researcher and PPI representative visited support groups (eg Diabetes Support Group, Heart Care Club) for recruitment purposes. The facilitator met with the participant on one occasion to deliver GENIE. The researcher was responsible for all the follow‐up data collection. Services employing lay health workers were approached to discuss involvement in the research, including training, identifying participants and intervention delivery. Facilitators comprised Health Trainers (n = 4), Care Navigators (n = 3), Community Navigators (n = 1) and Local Area Co‐ordinators (n = 1). A PPI representative and researchers (n = 3) were also trained.

## 4 RESULTS

Observational data provided a visual record of facilitation style, how participants related to facilitators and how comfortable they appeared physically. Notes taken on body language and gestures (eg leaning towards laptop screen, pointing to network map, positive response to visual cues) indicated that most participants felt at ease with the facilitator and engaged in the network mapping. Observations revealed that a natural balance of eye contact between participant, laptop and paperwork on the part of the facilitator helped to maintain engagement and co‐production (Box 1).

Box 1 **Example**

Instances of excessive focus on the laptop and paperwork captured evidence of disengagement, for example participant sitting back, distancing himself from the screen, folding his arms and vaping [HF10].

Facilitation is viewed as something that is initially co‐produced but orientated towards individual ownership of the network map and links to favoured activities. Three themes illuminated the social network mapping and preference linking processes:

1. Reversing the focus on the self and bringing others into view
2. Visualization and reflection as a mediator of positive disruption and linking to new connections
3. Personalized matching of valued activities a means of realizing preference elicitation

### 4.1 Reversing the focus on the self and bringing others into view

An exclusive focus on individuals' capacity and responsibility to initiate and sustain strategies for self‐management can leave those living with a LTC feeling stigmatized and labelled as unable to cope with daily activities. In contrast, co‐creation and engagement in network mapping provided a shared activity of both ‘doing’ and ‘interacting’ in which respondents began to reverse the focus on the ‘self’ and bring others into view as attention shifted towards relationships with the people around them. Refocusing away from self, towards other external, relevant sources of support (emotional, practical, physical, spiritual), helped to reduce the risk of acopia whilst increasing feelings of self‐worth.

This respondent shifted focus towards support derived from her daughter and pets, when recently widowed:

If it hadn't been for my daughter and the dog and cat I wouldn't have coped… I would probably have just crumbled into a little ball… (the dog) gets me out, gets me going for walks and is very loving. She shows me a lot of love…it's those stupid little sounding things that keep me going (HF14)

Face‐to‐face facilitation provided opportunities for respondents to cognitively re‐frame responsibility for LTC management from ‘individual’ to ‘shared’. Facilitators used name generating prompts to elicit successive recall of types of network members (eg friends, family, groups, pets), encouraging respondents to expand and diversify their networks:

If you are looking at it on a computer you think ‘Right, ok, what support have I got?’ and you wrack your brain trying to think but actually doing it face to face with somebody you can get prompted to think ‘Yes, there are more people’. So, then it's actually drawing on those resources, friends, relatives, GPs, pharmacist, whoever, to actually help you get what you need and the support you need (EU09)

Facilitator prompts were assistive aspects of co‐creating the network map:

I personally think it's better facilitated because there can be the odd prompt, ‘Where do you think it sits better there or there?’ And then underneath that, why? Because if you are sitting on your own you won't ask yourself those questions, you don't do you, unless you are particularly self‐aware? Some people will go through that mental checklist but most people won't (HF07)

…but I wouldn't have had all those names down on my own, I certainly wouldn't. I would have looked at it and said who is important? ‘Oh [stepdaughter 1] is’ and then I would be looking at… yes, the doctor is but I wouldn't have been, some of these round the outer ones, I wouldn't even dream that they were anything to do with it. So, it was… you need a bit of a prompt…my outlook has changed (EU11)

Conversations embedded in the network mapping activity enabled respondents to move from network member identification to reflect on social and emotional aspects of their relationships, environments and preferences. Facilitated conversation helped to ‘unpack’ thoughts and beliefs, highlighting the value of social relationships and practices as respondents considered the nature of support associated with particular individuals and groups:

Each one has a life of its own, it's not just a name or just a club. If I go back to one in particular, the Heart Care Club, there's 15 people there, each one of them I know. I've been going for six years now and I know about them all, I know about their families, I know about their problems, they know about my problems and that sharing of information is very powerful in terms of dealing with things and the inclination to keep it to yourself diminishes because we all, maybe it's a masculine thing (HF07)

Well it makes me think about things more if you know what I mean? You take a lot of things for granted and think well that's happening but unless you have them [facilitator]come along, I'd just have been accepting all these things but not putting them in any sort of position in my life. It's made me think more about what does [name] do for me?… or what does another person do for me? (EU11)

Relying on others and negotiating support was not always easy:

Well, it makes you think of different ways that you could do things and I suppose it makes you look at yourself as well… it makes you evaluate what people will do and what they won't do. I suppose because I've always been independent and rely on myself and not other people it's harder to rely on other people or ask them to help, to be quite honest (HF15)

Facilitated conversations around the network mapping exercise helped respondents let go of expectations from ‘strong’ ties (family) and value social connections with friends and acquaintances (weak ties). Expressing a perceived lack of family support (son had no time to help in the garden; limited contact with sisters), one respondent was able to re‐focus on the value of regular visits, shared time and activities with ‘more reliable’, long‐standing friendships. Similarly, this respondent was able to acknowledge the importance of ‘weak ties’/acquaintances in his everyday life:

I get an awful lot of support from people that most of… a lot of people take for granted. I don't take the ladies in Morrison's for granted… or the staff in the Co‐op… or bus drivers. I'm aware, but… a lot of people aren't that aware of that (HF.02)

Facilitation helped people reflect on the value of shared activities, replacing individual pursuits with joint activities. This respondent took up walking, which became a shared activity with her partner:

It may have been that the session that we did maybe made me more mindful of the fact that I needed to do more walking and stuff…I've done some walks, quite a few walks actually …and then we [partner and respondent] joined English Heritage and National Trust, so it went from there really. It was a gentle walking programme at work…I was keen to do the walking and found that that was really enjoyable, so I've stuck with the walking… (HF04)

### 4.2 Visualization and reflection as a mediator of positive disruption and linking to new connections

**Figure 1:**
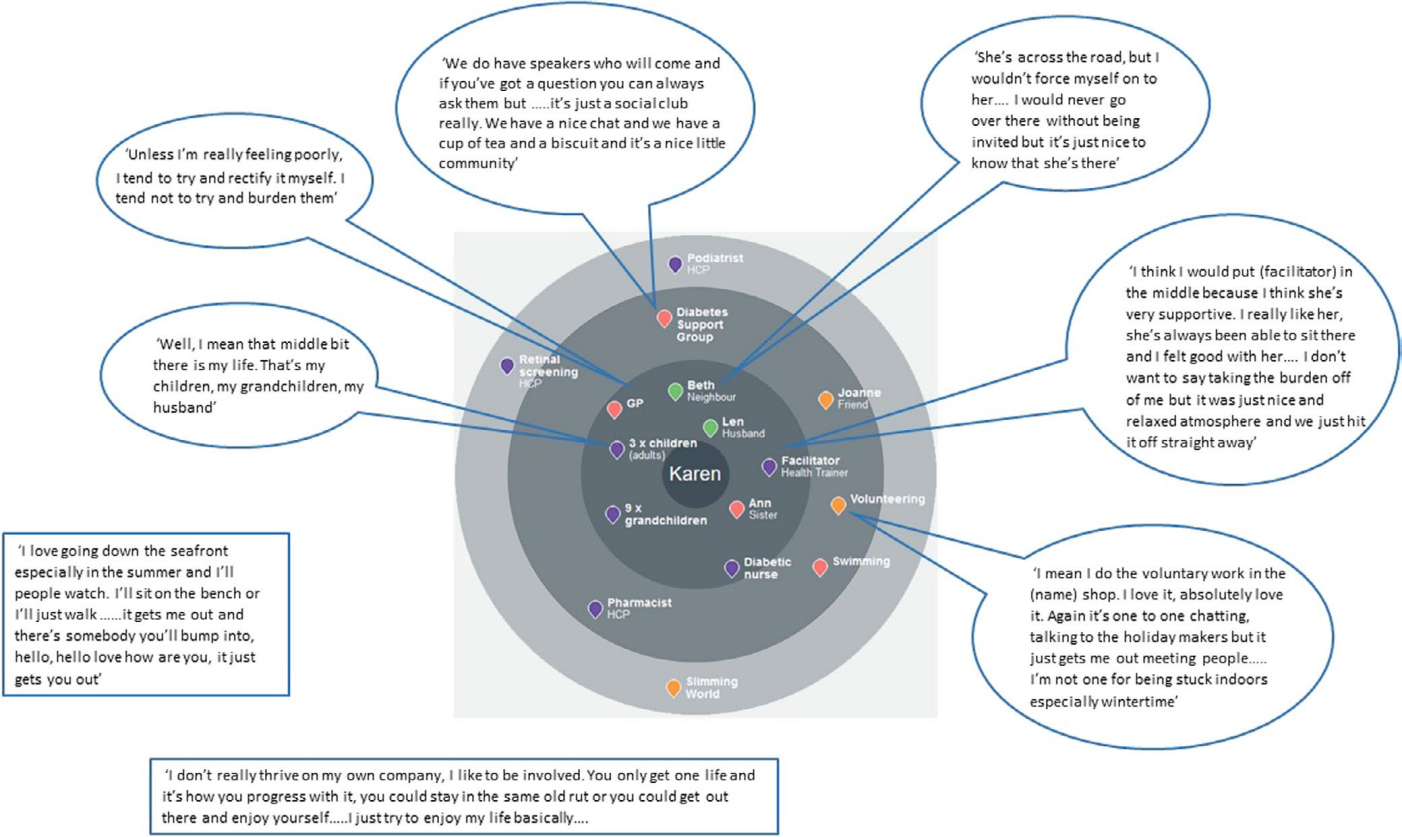
Co‐production of network map: participant reflections

Visualization and reflection on the completed network map enabled sense to be made of a novel means of support and how it could be accessed. Reflection on existing network membership opened up possibilities for evaluating the present and anticipating, rehearsing and reconstructing self‐management differently for the future (Figure 1).

This respondent acknowledged the value of visualization through reflecting on the meaning and value that new options for the future might bring in terms of socializing with others:

…a support network tends to go with going out socialising, because if you are not going out socialising as much then the support network is not necessarily there, do you know what I mean? So, I haven't been going out as much the last year because of my illness, so I don't tend to socialise much with people. So, I have been reflecting on that…, it did help me, just the visual…cos I'm a very visual learner, so a very visual thing has helped (HF04)

Rather than SMS being associated solely with the inner motivational life of an individual in relation to behaviour change, reflection on network support through visualization enabled normalization of SMS in everyday life and all it involves in terms of social environment, relationships and activities. The following respondent's terminology reflects this, referring to the collective ‘*we*’ and ‘getting on with *our* lives’, that is not ‘I’, getting on with ‘my’ life:

…it made me focus at a conscious level on the support infrastructure that I have available. And that made me feel good, because we're trying to get on with getting on, we're getting on with our lives and at an intellectual level you don't rationalise what's going on around you. You don't think about it, you sort of take it for granted. And I found the process enlightening, because it reminded me of how well supported I am. Which I have to say is very, very powerful. … it reminded me of what resources I have…it was a useful reminder of how many people there are out there that I can access if I need to (HF07)

Co‐creating a visual image of a person's support network seemed to enhance the process of identifying and triggering network members:

When you first start off, you start thinking, yes I've got my wife, I speak to my mum, I had Diabetes UK… but then everything else it's like opening up the flood gate! You don't realise…Oh yes, I have got things that I can do. I have got people that are around me'… it's actually knowing the amount of people that you've actually got around you that you can call on … I've got my healthcare trainer, I've got neighbours, podiatrist that I see every six weeks, it opens up a lot more…and yes I've got the next circle, yes I go out, I go to Church, I go to bowls, I can speak to my pharmacist and if I don't want to go down to the doctors I'll go down and see the pharmacist…so it opens up like a can of worms. …then the can is getting opened up more and more and then everything spills out and you find out you have got a lot more people around you that you can actually rely on (EU09)

This respondent commented on the visual design of the three concentric circles acting as an enabler for prioritizing individual network members:

When you actually think about, if you write it down on a list, you can't make sense of it … Doing it on there [network map], you're in the centre and everything works out from around you and when you think about it, like these are the people who are most important… You start thinking about it, pets, support groups, family all those things there. You can actually see it better if it's actually like a ripple going out. You see where the ones that are most important to you are in the centre near you and rippling out to who you can actually …so it is actually better having as a circle than just as a list because… It's actually prioritising who is most important to you and that circle gives the best way of doing it (EU09)

As respondents typically viewed self‐management as something they had to do on their own, many were surprised to see more network members in their completed diagram than they had anticipated:

With your health conditions you've got to look after number one and make sure that you are aware of what is going on… I do that on my own… It (circle diagram) was making sense. I just hadn't thought that…I hadn't thought about who was supporting me or who is supporting me…I'm surprised that… there are some people in the circle! (HF01)

### 4.3 Personalized matching of valued activities as a means of realizing preference elicitation

Facilitators guided respondents through an online preference questionnaire embedded within the intervention. Questions focused on what people valued doing or used to enjoy doing in the past. Rather than imposing a needs‐led framework, guiding people to identify what they enjoyed ensured activities were meaningful and relevant, increasing engagement in accessing community resources. Participants were guided by their own preference and identified 3 activities that most interested them. Most participants (n = 25) contacted/tried a new community resource, 2 participants did not take up activities and 3 participants withdrew (Kennedy, 2016; Vassilev, 2018). Follow‐up data indicated that participants enjoyed their chosen activities, suggesting a cognitive shift from thinking to doing.

Perceived lack of status difference between respondent and facilitator further increased engagement and proactivity on the part of the respondent. The lack of status difference was perceived intuitively from the observational data, as participants seemed at ease and open to working together with the facilitator.

Facilitated conversations represented a safe space for respondents to reflect on and express inner hopes and future wishes, enabling them to reconnect with the things that could bring enjoyment in everyday life. The following example indicates how a respondent living with mobility impairment shared his dream about experiencing an aqua‐lung in a swimming pool:

I haven't swum for a long, long time and when I did swim I did get some support and I did have somebody with me the whole time in the water…

Facilitator: Is that something you'd think about again if there was support?

… what I did dream of doing was having an aqua‐lung on my back, going underwater and just sitting on the bottom of the pool …just going into a pool or into a large fish tank, sit on the bottom of the tank and look at the fish going round and then stay there for about 5 minutes and then you come back up and that would have given me a lot of pleasure! You know, an achievement. Well, that's what I would like to do. I'd still like to do something like that – though I would take medical advice even now on that ‐ but that's one thing I would love to be able to do (EU02)

Aligning people's preferences and activity choices with their social connections, relationships and environment increased accessibility to activities and sustained engagement. However, findings also revealed social situations and environments that posed challenges. The following respondent was unable to remain in full‐time employment due to a LTC, but had identified a voluntary work opportunity which offered her purpose, social contact and fulfilment. Guided facilitation that took into account social and emotional aspects of everyday life enabled reflection on barriers to pursuing valued activities and future possibilities:

Yes, I love it (voluntary work) But now I can't go on Fridays because I'm at (partner's) mum's and I can't get there. I find it hard because I need somebody who will take me in the wheelchair. …But everything is on (partner's) time. It's when he wants to do it, when he's ready. … and I have to fit in. It's hard, so hard.

It's my lifeline really. … That's why I feel trapped at times. Not every day, not all the time but a lot of the time… I feel trapped because I can't… it's not like I can just get on the bus. I'm so dependent on (partner) (HF06)

The choice of focusing on lay health workers coalesced with the finding from a previous study that a lack of status difference between facilitator and participant was instrumental in allowing a stronger interpersonal element and reciprocity in the discussion to develop (Kennedy et al 2016). In this community‐based study, lay health workers met people in a place of their choice, usually at home. This participant comments on who is best placed to deliver the intervention and where this should take place:

Where's the best venue?… I think we'd have to go back to the home but with the caveat that they must have somebody with whom they feel comfortable…people do feel threatened, it's weird, isn't it? Why? I don't know… because that's people. It's got to be non‐threatening and in the conflicts context it was always a neutral ground, you went to the pub…but I'm just saying that was what you did. GPs represent authority, like it or not it's true, what I've been trained to believe (EU12)

## 5 DISCUSSION

Findings from the current study suggest that reversing the focus on self and bringing others into view enables a broader approach to SMS contrasting with traditional, narrower approaches that focus on individualized accountability and responsibility. This broader approach allows co‐production work initiated by the facilitator to move towards shared ownership, creating a space in which steer alternates between facilitator and respondent. As attention shifts from self (internal) to others (external), the fluid and flexible nature of this engagement opens up new possibilities for living and managing well that incorporate social relationships and societal participation. People living with a LTC are often forced to rely on their own resources. The SNI helped people to make a cognitive shift from individualized, self‐management accountability to being ready and receptive to the possibilities of collective effort. This finding resonates with existing research on the purpose of SMS. Morgan et al^8^ posited that the purpose of support in narrower approaches to SMS centred on individualized behavioural targets such as lifestyle, self‐monitoring and medication taking oriented to biomedical goals. Similarly, facilitation focuses on internal motivation of an individual rather than including those around them. A wider view and scope of facilitation explored here in a SNI highlights the need for a broader vision that incorporates other people, things and activities that are relevant to people's social contexts, life circumstances and lived experiences.

Findings demonstrate that facilitating visualization and reflection and expediting engagement with the network mapping exercise act to assist with positive disruption of established self‐management practices. Coherence and shift in perception of SMS from a process that focuses exclusively on the individual to one that incorporates a focus on collective elements and processes occur partly through the co‐creation of a visual image of a person's support network. All respondents entered into the shared network mapping activity with the facilitator and were able to reflect on its purpose in terms of sense making of how those around them operated as a, or potential, source of support or access to resources that would help with self‐management. This suggests that when the complexities of person's life world are presented in a visual format (concentric circles diagram), the information and its implications in terms of personal possibilities and challenges become more accessible, breaking down barriers to health inequality and improving health literacy. In relation to accessibility and sense making, Antonucci^22^ reported a similar finding when he first introduced the concentric circles hierarchical mapping technique to examine social support networks, commenting that the technique seemed to ‘transcend culture, age, life situation and crisis’. However, where the current study adds to this proven technique is by using the image of an existing support network to link into new social connections, imagine future possibilities and increase social participation. Moreover, recognition of people's life worlds and habitus as part of a broader entry into therapeutic landscapes has been highlighted as a means of living life well with a LTC.^26^

Empowerment and engagement were seemingly attained through the act of facilitation in which meaningful, enjoyable activities were identifiable and alignment could be made of valued preferences with people's social connections, relationships and environment in order to incorporate these into everyday life (Box 2).

Box 2 **Working example**

One participant had been recently diagnosed with diabetes T2 when she went through the intervention. One of the key things she wanted to do was to meet other local people with diabetes type 2 who were a similar age to herself (mid‐40s). Working through GENIE together with the facilitator, they identified a local, peer‐to‐peer support group for people recently diagnosed with diabetes T2, Sugarbuddies. Here, the participant reflects back during her 6‐month follow‐up interview:

‘I’m excited about Sugarbuddies. I feel that I'm getting more involved with it and they are people of my age group and by talking to them I understand what they're going through. I'm not on my own…it's not the end! There is life, you know, and it's positive compared to this time last year. What is the saying? Knowledge is power? It's opened up new doors for me…a new world – I'm very excited. There are very exciting times ahead!’ (EU01).

This process suggests that when people identify and are linked to valued activities that match what they enjoy doing, social participation is likely to increase in turn supporting people to manage better in everyday life. Reflecting on what people used to do in the past but no longer do is a powerful means of reintroducing the familiar and for people to reconnect with valued activities from the past.^3^ ^.^ It also acts as a counter to traditional approaches to SMS which identify with a predominant focus on education and advice‐giving where attention to psycho‐emotional issues patients face takes a back seat^14^ highlighting the need for facilitation to take into account the complexities of social circumstances and emotional responses of people. Implications for future training of health‐care professionals in self‐management point to the need for a basis of trust, questioning and flexibility.^14^ The current study points to how a SNA can incorporate the social, emotional and environmental needs required for creating a meaningful life in chronic illness management. In addition to relational aspects of facilitation, other aspects, such as awareness of locality, a sensitivity to and understanding of the bases of the value attributed to relationships and people's personal networks, collective community and social activities taking place locally, are relevant. Thus, a core element of future facilitation is the requirement of the development of a facilitator's empathy for the life worlds of individuals, in order that mutual empathy can lead to rapport and co‐appreciation of the preferences and choices being elicited and the links forged to others in a personal network. The latter is perhaps a salient quality to add to the facilitator‐patient relationship and addition to the lexicon of person‐centred interventions taking place in primary care and community settings.

### 5.1 Limitations

Data for this study comprised observations of the intervention delivery and qualitative, post‐intervention interviews with participants. Data collected directly from facilitators were limited to group feedback from lay health workers who took on the GENIE facilitator role for research purposes. Future research would benefit from conducting qualitative interviews with facilitators.

## 6 CONCLUSIONS

Our findings indicate that facilitating a SNI that includes a network mapping exercise as the key object of engagement requires an orientation towards the idea of connections and linkages and skills of enabling the exploration of relationships and interactions within people's everyday social world and requires an appreciation of the social environment and sense of place in order that the two people involved can work together authentically.

## CONFLICT OF INTEREST

The authors have no conflict of interest to declare.

## Data Availability

The data that support the findings of this study are available on request from the corresponding author. The data are not publicly available due to privacy or ethical restrictions. Access to anonymized data may be granted following review.
